# Adjuvant chemotherapy for colon carcinoma with positive lymph nodes: use and benefit in routine health care practice

**DOI:** 10.1054/bjoc.2001.2035

**Published:** 2001-09-01

**Authors:** C Bouchardy, P-E Queneau, G Fioretta, M Usel, M Zellweger, I Neyroud, L Raymond, C de Wolf, A P Sappino

**Affiliations:** Geneva Cancer Registry, Institute for Social and Preventive Medicine, 55 boulevard de la Cluse, Geneva, 1205, Switzerland; Department of Gastroenterology and Hepatology, University Hospital, Geneva, Switzerland; Department of Internal Medicine, University Hospital, Geneva, Switzerland; Division of Oncology, University Hospital, Geneva, Switzerland

**Keywords:** colon carcinoma, adjuvant chemotherapy, survival, good practices, patterns of care, cancer registry

## Abstract

In 1990, an international consensus was reached on the efficacy of adjuvant chemotherapy for lymph node positive (stage III) colon carcinoma (CC). This study evaluates the use and benefit of such therapy in routine health care practice. The study includes all patients with stage III CC treated by putative curative surgery (*n*= 182) recorded at the Geneva cancer registry between 1990 and 1996. Factors modifying chemotherapy use were determined by logistic regression, considering patients with chemotherapy as cases (*n*= 55) and others as controls (*n*= 127). The effect of chemotherapy on the 5-year survival was evaluated by the Cox model. Analyses were adjusted for possible confounders. The use of chemotherapy increased over the period (*P*_trend_ < 0.001). Age strongly modulated chemotherapy use. In 1996, 54% of eligible patients received chemotherapy, this proportion fell to 13% after age 70. Decisions to use chemotherapy significantly depended on stage, grade and cancer site. The chance to be treated was non-significantly lower among individuals of low social class, widowed and foreigners. Chemotherapy significantly decreased mortality rates (Hazard ratio: 0.35, 95%CI: 0.18–0.68), independently of the prognostic factors and with similar benefit regardless of stage and age group. Strong beneficial effect of adjuvant chemotherapy on stage III CC can be achieved in routine practice. However, this study shows that it is probably not optimally utilised in Switzerland, particularly among the elderly. © 2001 Cancer Research Campaign


*British Journal of Cancer* (2001) **85**(9), 1251–1257
© 2001 Cancer Research Campaign
doi: 10.1054/ bjoc.2001.2035, available online at http://www.idealibrary.com on IDEAL

### Adjuvant chemotherapy for colon carcinoma with positive lymph nodes: use and benefit in routine health care practice

**C Bouchardy[1], P-E Queneau[2], G Fioretta[1], M Usel[1], M Zellweger[3], I Neyroud[1], L Raymond[1], C de Wolf[1] and AP Sappino[4]**

[1]Geneva Cancer Registry, Institute for Social and Preventive Medicine, 55 boulevard de la Cluse, 1205 Geneva, Switzerland; [2]Department of Gastroenterology and Hepatology, University Hospital, Geneva, Switzerland; [3]Department of Internal Medicine, University Hospital, Geneva, Switzerland; [4]Division of Oncology, University Hospital, Geneva, Switzerland

**Summary** In 1990, an international consensus was reached on the efficacy of adjuvant chemotherapy for lymph node positive (stage III) colon carcinoma (CC). This study evaluates the use and benefit of such therapy in routine health care practice. The study includes all patients with stage III CC treated by putative curative surgery ($n$ = 182) recorded at the Geneva cancer registry between 1990 and 1996. Factors modifying chemotherapy use were determined by logistic regression, considering patients with chemotherapy as cases ($n$ = 55) and others as controls ($n$ = 127). The effect of chemotherapy on the 5-year survival was evaluated by the Cox model. Analyses were adjusted for possible confounders. The use of chemotherapy increased over the period ($P_{trend} < 0.001$). Age strongly modulated chemotherapy use. In 1996, 54% of eligible patients received chemotherapy, this proportion fell to 13% after age 70. Decisions to use chemotherapy significantly depended on stage, grade and cancer site. The chance to be treated was non-significantly lower among individuals of low social class, widowed and foreigners. Chemotherapy significantly decreased mortality rates (Hazard ratio: 0.35, 95%CI: 0.18–0.68), independently of the prognostic factors and with similar benefit regardless of stage and age group. Strong beneficial effect of adjuvant chemotherapy on stage III CC can be achieved in routine practice. However, this study shows that it is probably not optimally utilised in Switzerland, particularly among the elderly.
© 2001 Cancer Research Campaign

**Keywords**: colon carcinoma; adjuvant chemotherapy; survival; good practices; patterns of care; cancer registry

Colon carcinoma is one of the most frequent malignancies and one of the main causes of cancer deaths in industrialised countries. In Switzerland, it is estimated that each year approximately 1100 men and 1050 women are diagnosed with colon carcinoma (Levi et al, 1998). Despite the rather favourable prognosis, only half of the patients survive 5 years after diagnosis (Gatta et al, 1996; Berrino et al, 1999).

The only curative option for patients with colon carcinoma is surgery (Cohen et al, 1997). For stage I disease (Dukes' stage A and B-1 (Astler and Coller, 1954) invasion to the muscularis propria without nodal involvement) there is more than 90% probability of cure. This probability drops to approximately 75% for the stage II disease (Dukes' stage B-2, invasion into or through the serosa, without nodal involvement), and reaches only approximately 35% for the stage III disease (Dukes' stage C, metastasis to regional lymph nodes). The poor prognosis of nonmetastatic advanced disease is due to residual cancer in occult or microscopic form, for which chemotherapy or immunotherapy is most effective (Cohen et al, 1997).

At the end of the 1980s, randomised clinical trials provided evidence that adjuvant postoperative chemotherapy in colon carcinoma patients with regional lymph node metastasis increases the survival rates by approximately 30% (Laurie et al, 1989; Moertel et al, 1990; IMPACT, 1995). Data were less convincing for patients without positive lymph nodes.

Based on these evidences and given the infrequent toxic side effects, therapeutic guidelines were established in 1990, recommending systematic adjuvant chemotherapy after surgery for stage III colon carcinoma (NIH consensus conference, 1990). Recommendations are not always followed, and therefore great disparities may exist between guidelines and practice. There are nearly no data on the generalisation of such practice among the population and on the observed benefits outside clinical trials. This study evaluates the use and benefit of such therapy in routine health care practice in the Swiss canton of Geneva.

### MATERIALS AND METHODS

The data were derived from the Geneva cancer registry data set, which includes information on all incident cases of malignant neoplasms occurring in the population of the canton, approximately 400 000 inhabitants. The registration collects information from various sources and is considered accurate. This can be attested in particular by the very low percentage (< 2%) of cases recorded from death certificates only (Bouchardy, 1997). Notification is based on a voluntary agreement between the recording medical institutions of the canton and the registry. All hospitals, pathological laboratories and practitioners are requested to report all current and past cancer cases. Data are systematically abstracted from hospital and laboratory records by trained tumour

*Received 2 February 2001*
*Revised 11 June 2001*
*Accepted 4 July 2001*

*Correspondence to:* C Bouchardy registrars. Private practitioners regularly receive inquiry forms to secure missing clinical and therapeutic data. Death certificates are consulted systematically.

Recorded data include sociodemographic information (age, gender, nationality, place of birth, marital status and occupation), diagnostic circumstances (origin of diagnosis, presence of symptoms and methods of assessment), tumour characteristics (primary site, histologic type and differentiation coded according to the International Classification of Diseases for Oncology (ICD-O) ) (World Health Organization, 1976), stage of disease at diagnosis, treatment during the first 6 months after diagnosis (surgery, radiotherapy or chemotherapy), finality of treatment (curative, palliative or not specified), survival status and cause of death.

The Geneva cancer registry previously published a description of the survival assessment (Raymond et al, 1996). In brief, the index date for incidence refers to the confirmation date of diagnosis, or to the date of hospitalisation if it precedes the diagnosis and is related to the disease. The registry performs 2 types of follow-up. In addition to passive follow-up (routine examination of death certificates and hospital records), an active follow up is carried out routinely each year using the files of the Cantonal Population Office. For deceased patients, the cause of death is determined from clinical records and recorded systematically according to the World Health Organization classification (World Health Organization, 1967).

### Patient selection

The study was limited to colon carcinoma (ICD-O code 153). Cancers of the rectum and rectosigmoid junction (ICD-O codes 154.0 and 154.1) were not considered. Between 1990 and 1996, 930 patients with histologically confirmed first primary invasive colon carcinoma (excluding 4 malignant tumours other than carcinoma) were recorded in the resident population of the Swiss canton of Geneva. The study concerned only stage III colon cancer patients (Dukes' stage C, metastasis to regional lymph nodes) treated with putative curative surgery, i.e. surgery with total macroscopic and microscopic removal ($n$ = 182). We excluded patients who were not operated ($n$ = 3), or who had palliative surgery only ($n$ = 4) or surgery with no specification of being curative or not ($n$ = 9).

### Variables considered

The size of the tumour after resection (in cm) was classified in five categories (≤ 3, ≤ 4, ≤ 5, > 5 and unknown), and the T classification of the pathological pTNM classification (International Union against Cancer, 1992; 1987) in 4 groups: pT1 (submucosa) or pT2 (muscularis propria), pT3 (subserosa, non-peritonealized pericolic tissues), pT4 (visceral peritoneum/other organs structures) and pTx (unknown/unclassifiable). The level of lymph node invasion was studied using the pN classification in four groups: pN1 (≤ 3 pericolic), pN2 (> 3 pericolic), and pN3 (nodes on named vascular trunk). The tumour classification was coded according to the ICD-O code for histologic grading and differentiation: grade I (well differentiated, differentiated), grade II (moderately differentiated, moderately well differentiated), grade III (poorly differentiated), grade IV (undifferentiated, anaplastic) and unknown. Anatomical sites considered were: transverse colon (ICD-O 153.1), descending colon (ICD-O 153.2), sigmoid colon (ICD-O 153.3), caecum (ICD-O 153.4), ascending colon and appendix (ICD-O 153.5–6) and not specified (ICD-O 153.9).

4 levels of social class (based on the patient's last occupation or, for unemployed women, that of the spouse) were considered: *low* (manual employees, skilled and unskilled workers), *middle* (non-manual employees and administrative staff), *high* (professionals, executives, administrators) and *unknown*.

The health care sector in charge of the colon carcinoma surgery was of private (initial treatment in the private sector) or public nature (initial treatment in the public sector).

The methods of discovery (consultation following symptoms, screening or check-up examination, fortuitous discovery during consultation, unknown) were regrouped in 2 categories (screening, other). Screening procedures for colon carcinoma mainly referred to routine faecal occult blood testing or endoscopic examination.

Additional data on the type of chemotherapy among treated patients and on the presence of co-morbid conditions among untreated patients were collected from clinical files.

### Statistical analysis

### *Determinants of chemotherapy use*

Data were analysed through unconditional multivariate logistic regression, considering patients with adjuvant chemotherapy as cases and patients with no adjuvant chemotherapy as controls (Breslow and Day, 1980). All models were log-linear fitted using the generalised linear interaction modelling statistical package (Francis et al, 1993). The identified factors therefore concerned the modifiers of chemotherapy use. Factors of interest were alternatively age at diagnosis, gender, period of diagnosis, nationality, marital status, social class, method of discovery, health care sector, anatomical site, tumour differentiation, tumour size, T stage and N stage at diagnosis. The models contained the factor of interest and age (continuous) for estimation of the *crude effect*. For estimation of the adjusted effect, we a priori decided to adjust for all other variables linked to chemotherapy use. The significance of each variable of interest was assessed by comparing the goodness of fit measure (deviance according to degree of freedom) of the model with and without the variables of interest. Results are presented as relative risk estimates of being treated vs. untreated.

### *Estimation of the effect of adjuvant chemotherapy*

The 5-year survival was estimated by the actuarial method (intervals in months and standard error according to Greenwood). The effect of adjuvant chemotherapy on 5-year specific mortality rates was evaluated by Cox proportional hazards model accounting only for age (in continuous), or also for factors linked to both chemotherapy use and prognosis, such as tumour characteristics and stage (adjusted effect). Analyses were performed using both observed survival (total number of deaths) and specific survival (death from colon carcinoma only). In order to evaluate the potential variation of the effect of chemotherapy with individual or tumour characteristics, an interaction term involving chemotherapy use and age or T and N classifications, or tumour differentiation was introduced in the Cox model (Hill et al, 1990).

### RESULTS

Among the 182 colon carcinoma patients with positive lymph nodes who had curative surgery, 55 (30%) received adjuvant chemotherapy (the cases) and 127 did not (the controls). 89% of the treated group

 *© 2001 Cancer Research Campaign*

received the European standard therapy, i.e. 5-Fluorouracil plus Leucovorin (folinic acid).

### Determinants of adjuvant chemotherapy use

Table 1 shows the patient distribution according to sociodemographic characteristics and chemotherapy use. Chemotherapy use strongly diminished after the age of 70: approximately 50% of patients age ≤ 70 years received chemotherapy, compared with < 10% of patients age ≥ 70 years. After adjusting for confounders, the chance of being treated was more than 15-fold lower for patients ≥ 70 compared with those < 60 years (adjusted OR: 0.06, 95% CI: 0.02–0.18).

For 69 of the 85 patients aged ≥ 70 years who did not receive chemotherapy, clinical information on the existence or absence of co-morbid conditions was available. Of those 69 patients, 22 had co-morbid conditions (6 alcoholic, psychiatric or nervous system disorders; 5 pulmonary or cardiac disorders, 3 post-surgical complications; 8 other unfavourable general conditions such as cachexia, diabetes mellitus). For 47 patients no relevant co-morbid conditions were reported, and their general status was described as good. Among them, 2 patients refused treatment. For the others, age was mentioned to be the main reason for the clinical decision not to treat with chemotherapy. A typical sentence in the medical records was 'Due to the age of the patient, no adjuvant therapy was proposed'.

The proportion of treated patients increased over time. In 1992, less than 20% of the patients were treated with chemotherapy. This proportion increased to 54% in 1996. The probability of being treated was about 8-fold higher in 1995–1996 compared with the period 1990–1992 (adjusted OR: 7.74, 95% CI: 2.52–23.76).

With respect to other sociodemographic factors, widowed had approximately a 3-fold lesser chance of being treated compared with married individuals (adjusted OR: 0.34, 95% CI: 0.08–1.26). The chance to be treated was also lower for foreigners than for Swiss nationals (adjusted OR: 0.65, 95% CI: 0.22–1.97), and approximately 2-fold higher among individuals belonging to middle or high social class (adjusted OR: 2.42, 95% CI: 0.84–7.02 and OR: 1.84, 95% CI: 0.52–6.50, respectively). However, the effects of these factors were not significant. No gender difference was seen. In the current series, 6% of the stage III colon carcinoma were diagnosed by screening (Table 2). There was a significant link between method of discovery and chemotherapy use only in non-adjusted analyses (Table 2).

Approximately one third of the patients had surgery in private institutions. The proportion of patients with adjuvant chemotherapy was 41% in the private sector compared with 24% in the public sector. This reflected the differences in age (mean age of the

**Table 1** Distribution of stage III colon carcinoma patients and estimation of the effect of sociodemographic characteristics on adjuvant chemotherapy use

| | Adjuvant chemotherapy | | Crude effect | | Adjusted effect | |
|---|---|---|---|---|---|---|
| | Yes (cases) *n* = 55 | No (controls) *n* = 127 | OR[a] | (95%CI) | OR[b] | (95%CI) |
| Age group (years) | | | | | | |
| < 60 | 23 | 22 | 1[c] | | 1[c] | |
| 60–69 | 23 | 20 | 1.10 | (0.48–2.54) | 0.72 | (0.27–1.94) |
| ≥ 70 | 9 | 85 | 0.10*** | (0.04–0.25) | 0.06*** | (0.02–0.18) |
| Gender | | | | | | |
| Male | 29 | 62 | 1[c] | | 1[c] | |
| Female | 26 | 65 | 0.78 | (0.38–1.61) | 0.62 | (0.27–1.44) |
| Period of diagnosis | | | | | | |
| 1990–1992 | 14 | 61 | 1[c] | | 1[c] | |
| 1993–1994 | 17 | 40 | 1.78 | (0.71–4.43) | 2.28 | (0.78–6.68) |
| 1995–1996 | 24 | 26 | 4.88*** | (1.93–12.3) | 7.74*** | (2.52–23.76) |
| Civil status | | | | | | |
| Married | 35 | 65 | 1[c] | | 1[c] | |
| Widowed | 4 | 36 | 0.48 | (0.14–1.59) | 0.34 | (0.08–1.26) |
| Single | 8 | 14 | 0.89 | (0.36–2.60) | 0.68 | (0.19–2.50) |
| Other | 8 | 12 | 1.54 | (0.52–4.56) | 1.69 | (0.44–6.51) |
| Nationality | | | | | | |
| Swiss | 44 | 105 | 1[c] | | 1[c] | |
| Other | 11 | 22 | 0.41 | (0.15–1.14) | 0.65 | (0.22–1.97) |
| Social class | | | | | | |
| Low | 10 | 43 | 1[c] | | 1[c] | |
| Middle | 32 | 52 | 2.06 | (0.84–5.04) | 2.42 | (0.84–7.02) |
| High | 12 | 23 | 1.94 | (0.66–5.72) | 1.84 | (0.52–6.50) |
| Unknown | 1 | 9 | Excluded | | Excluded | |

[a]Odds ratio adjusted for age (continuous); [b]Odds ratio adjusted for age (continuous), period (continuous), cancer sub-site (caecum, other), differentiation (well, other), lymph node classification (N1, N2, other) and tumour classification (T1 and T2, T3, other); [c]Reference category; *$P < 0.05$, **$P < 0.01$, *** $P < 0.001$.

© 2001 Cancer Research Campaign 

**Table 2** Distribution of stage III colon carcinoma patients and estimation of the effect of method of discovery, and health care sector on adjuvant chemotherapy use

| | Adjuvant chemotherapy | | Crude effect | | Adjusted effect | |
|---|---|---|---|---|---|---|
| | Yes (cases) *n* = 55 | No (controls) *n* = 127 | OR[a] | (95%CI) | OR[b] | (95%CI) |
| Method of discovery | | | | | | |
| Other | 49 | 117 | 1[c] | | 1[c] | |
| Screening | 6 | 5 | 4.21* | (1.05–16.89) | 5.23 | (0.97–28.26) |
| Unknown | 0 | 5 | Excluded | | Excluded | |
| Health care sector[d] | | | | | | |
| Public | 27 | 86 | 1[c] | | 1[c] | |
| Private | 28 | 41 | 1.05 | (0.49–2.25) | 1.16 | (0.46–2.94) |

[a]Odds ratio adjusted for age (continuous); [b]Odds ratio adjusted for age (continuous), period (continuous), cancer sub-site (caecum, other), differentiation (well, other), lymph node classification (N1, N2, other) and tumour classification (T1 and T2, T3, other); [c]Reference category; [d]First treatment; $P < 0.05$, ** $P < 0.01$, ***$P < 0.001$.

patients privately treated was 64 years vs. 73 years for the public sector) and the chance of being treated was similar in both private and public practice after accounting for age (Table 2).

Table 3 presents the patient distribution by site and stage of the primary tumour. Caecum cancer was less frequently treated by chemotherapy than cancers of other sites (21% vs. 32%, $P = 0.20$) (Table 3). Caecum carcinoma was treated about 5 times less compared with transverse colon carcinoma (adjusted OR: 0.23, 95% CI: 0.05–0.99) (Table 3). The proportion of treated patients increased according to the level of tumour differentiation, with an almost 12-fold increase in use of chemotherapy for poorly differentiated tumours compared with well differentiated tumours (adjusted OR: 12.55, 95% CI: 2.16–73.07).

In the current series, 69% of the patients had less than 3 pericolic nodes invasion (N1), while 9% had a nodal invasion on named vascular trunk (N3). The probability of being treated was not significantly higher for N3 compared with N1 stages (adjusted OR: 2.37, 95% CI: 0.59–9.50). However, it was significantly lower when the tumour invaded through the muscularis propria into subserosa, or into pericolic tissues (T3 or T4), compared with lesser local invasion (T1 and T2), even after accounting for the severity of nodal invasion (OR: 0.16, 95% CI: 0.03–0.86 and OR: 0.10, 95% CI: 0.02–0.42 for T4 and T3, respectively). The tumour size had no influence on chemotherapy use.

### Effect of adjuvant chemotherapy

After 5 years, 85 patients were still alive, 74 patients died of colon carcinoma and 21 died of other causes. 2 patients were lost to follow-up because they left the canton. The mean follow-up was 1138 days (standard deviation: 644). The 5-year survival of colon carcinoma patients with positive lymph nodes and curative surgery was 45% (standard error: 4%) for crude survival and 54% (standard error 4%) for specific survival. Since results on crude and specific survival were very similar, we decided to present only the observed survival data. Table 4 shows the effect of potential prognostic factors on instantaneous death rates. Results were adjusted for age, or for age plus period, T and N classifications and tumour site. Age had no effect on prognosis when accounting for other prognostic factors, while the effect of period and N stage remained significant.

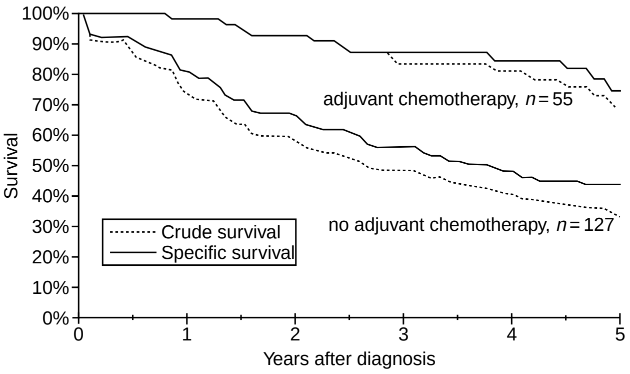

**Figure 1** Survival after curative surgery for stage III colon carcinoma

Regardless of age, period, site, T and N classifications, the patients who received chemotherapy had an almost 3-fold (Hazard ratio: 0.35, 95% CI: 0.18–0.68) significantly decreased mortality rate. Adjustment for additional factors, such as social class and method of discovery, did not modify the results. None of the interaction analyses was significant, i.e. the effect of chemotherapy was similar regardless of age-group, stage or tumour differentiation.

As observed in Figure 1, the 5-year survival was higher in the groups treated vs. untreated with adjuvant chemotherapy, for both observed survival (70%, standard error: 5% vs. 34%, standard error 3%), and specific survival (75%, standard error: 7% vs. 44%, standard error 5%).

Only patients who survived long enough could undergo chemotherapy treatment, so additional analyses were performed on the same series, excluding 18 patients who died during the first 6 months after diagnosis. As expected, all these patients belonged to the untreated group.

Nevertheless, these new analyses provided similar results for both determinants of chemotherapy use and efficacy of chemotherapy. The hazard ratio associated with the use of chemotherapy in multi-adjusted Cox model was 0.37 (95% CI: 0.18–0.73), i.e. very close to the results presented in Table 4.

 *© 2001 Cancer Research Campaign*

**Table 3** Distribution of stage III colon carcinoma patients and estimation of the effect of tumour characteristics on adjuvant chemotherapy use

| | Adjuvant chemotherapy | | Crude effect | | Adjusted effect | |
|---|---|---|---|---|---|---|
| | Yes (cases) *n* = 55 | No (controls) *n* = 127 | OR[a] | (95%CI) | OR[b] | (95%CI) |
| Anatomical site | | | | | | |
| Transverse colon | 10 | 31 | 1[c] | | 1[c] | |
| Descending colon | 4 | 7 | 0.93 | (0.16–5.14) | 0.53 | (0.08–3.42) |
| Sigmoid colon | 25 | 33 | 1.42 | (0.53–3.79) | 1.38 | (0.44–3.31) |
| Caecum | 7 | 26 | 0.35 | (0.09–1.27) | 0.23* | (0.05–0.99) |
| Ascending colon[d] | 9 | 21 | 0.57 | (0.17–1.98) | 0.53 | (0.13–2.11) |
| Not specified | 0 | 9 | Excluded | | Excluded | |
| Tumour differentiation | | | | | | |
| Well | 5 | 18 | 1[c] | | 1[c] | |
| Moderately | 31 | 87 | 2.05 | (0.58–7.21) | 2.37 | (0.56–9.92) |
| Poorly | 17 | 14 | 10.26** | (2.35–44.84) | 12.55** | (2.16–73.07) |
| Unknown | 2 | 8 | Excluded | | Excluded | |
| Size of tumour (cm) | | | | | | |
| ≤ 3 | 13 | 26 | 1[c] | | 1[c] | |
| ≤ 4 | 14 | 41 | 0.65 | (0.23–1.82) | 0.46 | (0.14–1.52) |
| ≤ 5 | 16 | 24 | 1.03 | (0.35–3.02) | 0.95 | (0.27–3.34) |
| ≥ 6 | 10 | 34 | 0.43 | (0.14–1.32) | 0.60 | (0.17–2.16) |
| Unknown | 2 | 2 | Excluded | | Excluded | |
| TNM classification | | | | | | |
| T1 | 1 | 1 | ] 1[c] (T1–T2) | | ] 1[c] (T1–T2) | |
| T2 | 7 | 6 | | | | |
| T3 | 40 | 99 | 0.33 | (0.10–1.14) | 0.10** | (0.02–0.42) |
| T4 | 7 | 19 | 0.28 | (0.06–1.28) | 0.16* | (0.03–0.86) |
| Unknown | 0 | 2 | Excluded | | Excluded | |
| TNM classification | | | | | | |
| N1 | 33 | 92 | 1[c] | | 1[c] | |
| N2 | 15 | 22 | 1.40 | (0.57–3.40) | 1.03 | (0.36–2.91) |
| N3 | 7 | 10 | 3.21 | (0.93–11.05) | 2.37 | (0.59–9.50) |
| Unknown | 0 | 3 | Excluded | | Excluded | |

[a]Odds ratio adjusted for age (continuous); [b]Odds ratio adjusted for age (continuous), period (continuous), cancer sub-site (caecum, other), differentiation (well, other), lymph node classification (N1, N2, other), and tumour classification (T1 and T2, T3, other); [c]Reference category; [d]Appendix included; * $P < 0.05$, ** $P < 0.01$, *** $P < 0.001$.

### DISCUSSION

This study shows that in daily practice the probability of receiving adjuvant chemotherapy after surgery for stage III colon carcinoma remains low, despite established recommendations. According to the literature, the expected proportion of ineligible patients should be round 5% (Moertel et al, 1990; NIH Consensus conference 1990; IMPACT, 1995), whereas we observed that still in 1996 practically 50% of the patients were not treated.

We found very few studies providing population data on adjuvant chemotherapy use for stage III colon carcinoma in general practice after the publication of the therapeutic guidelines in the 1990s (NIH consensus conference, 1990). The proportion of treated patients was 35% in a French study in 1994–1995 (Jouve et al, 1998) and 51% in a New Jersey study in 1989–1996 (Mahoney et al, 2000). Adjuvant chemotherapy administration strongly decreases with age. In 1996, < 13% of patients age ≥ 70 years received adjuvant chemotherapy. This could reflect the decline of the general condition due to old age and a higher prevalence of co-morbid conditions, or a patient's refusal. But also it could be the consequence of a general unwillingness to treat the elderly, possibly due to the limited data on chemotherapy efficacy among patients age ≥ 70 years (Trimble et al, 1994). Co-morbidity was absent in about 2/3 of the patients aged ≥ 70 who did not receive chemotherapy. In this study, the information collected on co-morbid conditions was done retrospectively and is probably not complete. However, the results are compatible with those previously reported (Jouve et al, 1998; Mahoney et al, 2000), and strongly suggest that the age factor per se is limiting chemotherapy prescription.

Recent data provided reassuring evidence on the tolerance, as well as on the efficacy of chemotherapy among elderly patients with colon carcinoma (Ross et al, 1998; Popescu et al, 1999). Arbitrary age cut-off appears therefore unjustified. The current study does not show any significant differences in chemotherapy efficacy between age-groups ($\chi^2$ interaction test between age and chemotherapy = 5.6, $P = 0.13$). In particular, among the 9 patients age ≥ 70 years who received chemotherapy, all except 1 were alive after 5 years. Because cancer occurs more often in the elderly (in this study, one fourth of stage III colon cancer concerned patients age ≥ 70 years), the indication for chemotherapy among this group is a public health matter that should not be neglected. The adjuvant

*© 2001 Cancer Research Campaign* 

**Table 4** Effect of adjuvant chemotherapy and prognostic factors on instantaneous mortality rates after surgery for stage III colon carcinoma

| | Cox proportional hazards model accounting for age only | | | | Cox proportional hazards model accounting for other prognostic factors | |
|---|---|---|---|---|---|---|
| | **Cases** *n* **= 182** | **Deaths** *n* **= 95** | **Hazard ratio**[a] | **(95% CI)** | **Hazard ratio**[b] | **(95%CI)** |
| Chemotherapy | | | | | | |
| No | 127 | 81 | 1[c] | | 1[c] | |
| Yes | 55 | 14 | 0.37** | (0.20–0.68) | 0.35** | (0.18–0.68) |
| Age-group (years) | | | | | | |
| < 60 | 45 | 18 | 1[c] | | 1[c] | |
| 60–69 | 43 | 18 | 1.20 | (0.62–2.30) | 1.54 | (0.78–3.02) |
| ≥ 70 | 94 | 59 | 2.13** | (1.25–3.61) | 1.68 | (0.95–2.97) |
| Period of diagnosis | | | | | | |
| 1990–1992 | 75 | 49 | 1[c] | | 1[c] | |
| 1993–1994 | 57 | 30 | 0.76 | (0.48–1.20) | 0.77 | (0.47–1.26) |
| 1995–1996 | 50 | 16 | 0.49* | (0.28–0.87) | 0.51 | (0.27–0.96) |
| Anatomical site | | | | | | |
| Transverse colon | 41 | 20 | 1[c] | | 1[c] | |
| Descending colon | 11 | 4 | 0.87 | (0.30–2.54) | 0.68 | (0.21–2.22) |
| Sigmoid colon | 58 | 28 | 1.48 | (0.82–2.66) | 1.55 | (0.84–2.85) |
| Caecum | 33 | 20 | 1.72 | (0.92–3.21) | 1.52 | (0.80–2.88) |
| Ascending colon | 30 | 18 | 1.90 | (1.00–3.61) | 1.70 | (0.87–3.31) |
| Not specified | 9 | 5 | | Excluded | | Excluded |
| TNM classification | | | | | | |
| T1, T2 | 15 | 6 | 1[c] | | 1[c] | |
| T3 | 139 | 71 | 1.36 | (0.59–3.14) | 0.98 | (0.40–2.36) |
| T4 | 26 | 18 | 2.12 | (0.84–5.36) | 1.43 | (0.55–3.73) |
| Unknown | 2 | – | | Excluded | | Excluded |
| TNM classification | | | | | | |
| N1 | 125 | 60 | 1[c] | | 1[c] | |
| N2 | 37 | 23 | 2.32** | (1.39–3.87) | 2.51*** | (1.50–4.21) |
| N3 | 17 | 12 | 1.77 | (0.95–3.30) | 2.50** | (1.29–4.86) |
| Unknown | 3 | – | | Excluded | | Excluded |

[a]Hazard ratio adjusted for age (continuous); [b]Hazard ratio adjusted for age (continuous), chemotherapy (no, yes), period (continuous), site of the tumour (caecum, other), T classification (T1 and T2, T3, T4, unknown), N classification (N1, N2, N3, unknown); [c]Reference category; *$P < 0.05$, **$P < 0.01$, ***$P < 0.001$.

chemotherapy use was higher among patients with poorly differentiated tumours, indicating a prescription targeting patients presenting carcinogenic criteria associated with a worse prognosis. However, there are no real indications against treating patients with well or moderately differentiated tumours. With regard to local invasion, there was a lesser propensity to treat tumours invading through the muscularis propria when compared with more favourable stages. This was also observed for caecal cancer, which is often diagnosed at a more advanced stage, probably due to its possibility to extend before occurrence of symptoms such as occlusion (Cohen et al, 1997). The worse general condition and the obstructive presentation (Wolmark et al, 1983) are likely to explain the lesser probability of being treated when the local invasion increases. There was a general tendency of reduced chemotherapy use among widowed, foreigners and patients belonging to lower socioeconomic class, demonstrating once more that the disfavoured have a lower access to optimal treatment (Polednak, 1989). These last results were not significant, probably because of the low power of the study. Reassuringly, the propensity of being treated was similar in private institutions and the university hospital sector, even after an additional adjustment for socioeconomic status (OR: 1.1; 95%CI: 0.5–2.7).

Beyond clinical trials, we have hardly any information on the effect of adjuvant chemotherapy in routine health care practice. With regard to data available in current practice derived from the US national cancer data base, patients with stage III carcinoma diagnosed between 1985–1993 were found to have an increase of 3% of the 3-year relative survival (Jessup et al, 1996). That study, however, involves prescriptions before 1990, a period when various drugs were used without proven effectiveness. In other studies, effectiveness of chemotherapy in stage III carcinoma was not reported (Beart et al, 1995; Jouve et al, 1998).

We observed a relative reduction in death rates of 65% among treated patients (95% CI: 32–82%), i.e. approximately twice that expected from clinical trials (Moertel et al, 1990; Wolmark et al, 1993; Francini et al, 1994; IMPACT, 1995; O'Connell et al, 1997; Mamounas et al, 1999). This important difference in survival is not generated from a randomised study, and partly reflects the lower propensity to give chemotherapy to patients with putative poorer prognosis. In current practice the choice, whether to treat or not, is based on the presence of co-morbid conditions: the observed 5-year survival in untreated patients was 34% compared to approximately 43% in control groups in clinical trials (Laurie et al, 1989; Francini et al, 1994). The 5-year survival in treated patients (70%)

 *© 2001 Cancer Research Campaign*

is very close to that observed in the treated group in clinical trials (approximately 70%) (Laurie et al, 1989; Francini et al, 1994). To minimise the effect of patient selection we excluded all patients who died within 6 months of diagnosis, and obtained a very similar result of chemotherapy effectiveness.

### CONCLUSION

Adjuvant chemotherapy has proved its effectiveness for stage III colon carcinoma patients, however, it has not reached its full potential in daily practice. The probability of being treated remains low, particularly among the elderly. This non-randomised study based on a relatively small group of patients confirms the beneficial effect of adjuvant chemotherapy use in routine practice.

### ACKNOWLEDGEMENTS

The authors thank Prof J Torhorst and Prof U Laffer for initiating this study and Mrs Stina Blagojevic for her expert technical help and editorial assistance.

### REFERENCES

Astler VB and Coller FA (1954) The prognostic significance of direct extension of carcinoma of the colon and rectum. *Ann Surg* **139**: 846

Beart RW, Steele GDJ, Menck HR, Chmiel JS, Ocwieja KE and Winchester DP (1995) Management and survival of patients with adenocarcinoma of the colon and rectum: a national survey of the Commission on Cancer. *J Am Coll Surg* **181**: 225–236

Berrino F, Capocaccia R, Esteve J, Gatta G, Hakulinen T, Micheli A, Sant M and Verdecchia A (1999) *Survival of cancer patients in Europe: the EUROCARE-2 study*. Lyon, International Agency for Research on Cancer. IARC Scientific Publications N˚ 151

Bouchardy C (1997) Switzerland, Geneva. In: *Cancer incidence in five continents. Vol. VII*, Parkin DM, Whelan SL, Ferlay J, Raymond L, Young J (eds) pp 666–669. International Agency for Research on Cancer: Lyon

Breslow NE and Day NE (1980) Statistical methods in cancer research. Volume 1 – The analysis of case-control studies. Lyon, International Agency for Research on Cancer. IARC Scientific Publications N˚ 32

Cohen AM, Minsky BD and Schilsky RL (1997) Cancer of the colon. In *Cancer Principles & Pratice of Oncology*, DeVita VT, Hellman S, Rosenberg SA (eds) pp 1144–1197. Lippincott-Raven: Philadelphia

Francini G, Petrioli R, Lorenzini L, Mancini S, Armenio S, Tanzini G, Marsili S, Aquino A, Marzocca G and Civitelli S (1994) Folinic acid and 5-fluorouracil as adjuvant chemotherapy in colon cancer. *Gastroenterology* **106**: 899–906

Francis B, Green M and Payne C (eds) (1993) GLIM 4 the statistical system for generalized linear interactive modelling. Oxford University Press: Oxford

Gatta G, Sant M, Coebergh JW and Hakulinen T (1996) Substantial variation in therapy for colorectal cancer across Europe: EUROCARE analysis of cancer registry data for 1987. *Eur J Cancer* **32A**: 831–835

Hill C, Com-Nougué C, Kramar A, Moreau T, O'Quigley J, Senoussi R and Chastang C (1990) Les modèles de survie en recherche clinique. In *Analyse statistique des données de survie*, INSERM (ed) pp 129–143. Flammarion: Paris

IMPACT, International Multicentre Pooled Analysis of Colon Cancer Trials investigators (1995) Efficacy of adjuvant fluorouracil and folinic acid in colon cancer. *Lancet* **345**: 939–944

International Union against Cancer UICC (1987) TNM classification of malignant tumours. 4th edition. Springer-Verlag: Berlin

International Union against Cancer UICC (1992) TNM Classification of malignant tumours. 4th edition, 2nd revision. Springer Verlag: Berlin

Jessup JM, McGinnis LS, Steele GDJ, Menck HR and Winchester DP (1996) The National Cancer Data Base. Report on colon cancer. *Cancer* **78**: 918–926

Jouve JL, Mitry E, Phelip JM, Villing AL, Tazi MA and Faivre J (1998) Chimiothérapie adjuvante pour adénocarcinome colique dans le département de la Côte-d'Or. *Gastroenterol Clin Biol* **22**: 269–272

Laurie JA, Moertel CG, Fleming TR, Wieand HS, Leigh JE, Rubin J, McCormack GW, Gerstner JB, Krook JE and Malliard J (1989) Surgical adjuvant therapy of large-bowel carcinoma: an evaluation of levamisole and the combination of levamisole and fluorouracil. The North Central Cancer Treatment Group and the Mayo Clinic. *J Clin Oncol* **7**: 1447–1456

Levi F, Raymond L, Schüler G, Fisch T, Bouchardy C, Allemann J, Joris F and Torhorst J (1998) *Cancer en Suisse. Faits et commentaires*. Ligue Suisse contre le Cancer: Berne

Mahoney T, Kuo YH, Topilow A and Davis JM (2000) Stage III colon cancers: why adjuvant chemotherapy is not offered to elderly patients. *Arch Surg* **135**: 182–185

Mamounas E, Wieand S, Wolmark N, Bear HD, Atkins JN, Song K, Jones J and Rockette H (1999) Comparative efficacy of adjuvant chemotherapy in patients with Dukes' B versus Dukes'C colon cancer: results from four National Surgical Adjuvant Breast and Bowel Project adjuvant studies (C-01, C-02, C-03, and C-04). *J Clin Oncol* **17**: 1349–1355

Moertel CG, Fleming TR, Macdonald JS, Haller DG, Laurie JA, Goodman PJ, Ungerleider JS, Emerson WA, Tormey DC and Glick JH (1990) Levamisole and fluorouracil for adjuvant therapy of resected colon carcinoma. *N Engl J Med* **322**: 352–358

NIH consensus conference (1990). Adjuvant therapy for patients with colon and rectal cancer. *Jama* **264**: 1444–1450

O'Connell MJ, Mailliard JA, Kahn MJ, Macdonald JS, Haller DG, Mayer RJ and Wieand HS (1997) Controlled trial of fluorouracil and low-dose leucovorin given for 6 months as postoperative adjuvant therapy for colon cancer. *J Clin Oncol* **15**: 246–250

Polednak A (1989) *Racial and ethnic differences in disease*. Oxford University Press: New York

Popescu RA, Norman A, Ross PJ, Parikh B and Cunningham D (1999) Adjuvant or palliative chemotherapy for colorectal cancer in patients 70 years or older. *J Clin Oncol* **17**: 2412–2418

Raymond L, Fischer B, Fioretta G and Bouchardy C (1996) Migration bias in cancer survival rates. *J Epidemio Biostatistics* **1(3)**: 167–173

Ross PJ, Popescu RA, Cunningham D, Norman A and Parikh B (1998) * 1069 Adjuvant and palliative chemotherapy for colorectal cancer in patients aged 70 years or older. *ASCO* **17**: 278A

Trimble EL, Carter CL, Cain D, Freidlin B, Ungerleider RS and Friedman MA (1994) Representation of older patients in cancer treatment trials. *Cancer* **74**: 2208–2214

Wolmark N, Wieand HS, Rockette HE, Fisher B, Glass A, Lawrence W, Lerner H, Cruz AB, Volk H and Shibata H (1983) The prognostic significance of tumor location and bowel obstruction in Dukes B and C colorectal cancer. Findings from the NSABP clinical trials. *Ann Surg* **198**: 743–752

Wolmark N, Rockette H, Fisher B, Wickerham DL, Redmond C, Fisher ER, Jones J, Mamounas EP, Ore L and Petrelli NJ (1993) The benefit of leucovorin-modulated fluorouracil as postoperative adjuvant therapy for primary colon cancer: results from National Surgical Adjuvant Breast and Bowel Project protocol C-03. *J Clin Oncol* **11**: 1879–1887

World Health Organization (WHO) (1967) International classification of diseases, 1965 revision. World Health Organization: Geneva

World Health Organization (WHO) (1976) ICD-O: International classification of diseases for oncology. 1st edition. World Health Organization: Geneva

*© 2001 Cancer Research Campaign*
